# FasterGeo-YOLO: Multi-Scale Geometric Adaptive Feature Enhancement for Lightweight Road Damage Detection

**DOI:** 10.3390/s26206619

**Published:** 2026-10-19

**Authors:** Longshuai Zhang, Ru Yang, Zhentao Qin, Yuanjie Lu, Boyi Zhang, Shuqi He, Yuwei Jin

**Affiliations:** 1 School of Mathematics and Computer Science, Panzhihua University, Panzhihua 617000, China; lifeyu421@gmail.com (L.Z.); qinzhentao@pzhu.edu.cn (Z.Q.); gioockby@gmail.com (B.Z.); heshuqi_2002@foxmail.com (S.H.); 2 School of Civil and Architecture Engineering, Panzhihua University, Panzhihua 617000, China; 3 School of Electrical and Information Engineering, Panzhihua University, Panzhihua 617000, China; 19856492280@139.com (Y.L.); jinyuwei@alu.uestc.edu.cn (Y.J.)

**Keywords:** road damage detection, lightweight object detection, geometric adaptive enhancement, deformable convolution, high-resolution detail compensation

## Abstract

Automatic road damage detection is essential for intelligent transportation infrastructure inspection and road health monitoring. However, road damage targets often exhibit small scales, weak boundaries, irregular geometries, low texture contrast, and large inter-class appearance variations, which make accurate and efficient detection challenging for lightweight detectors. To address these issues, this paper proposes FasterGeo-YOLO, a lightweight multi-scale geometric adaptive enhancement framework based on the YOLO26n baseline. FasterNet is introduced as an efficiency-oriented lightweight backbone, while C2f_DCN deformable convolution modules are embedded at the P3 and P4 feature levels to provide complementary geometric adaptation at different spatial resolutions. The original YOLO26n baseline is DFL-free, whereas FasterGeo-YOLO intentionally reintroduces Distribution Focal Loss (DFL) in its modified detection formulation to strengthen distribution-based bounding-box regression. For fair baseline comparison, all detectors in the main comparison are evaluated at 640 × 640 input resolution; the subsequent 800 × 800 stage is treated separately as a high-resolution refinement strategy. At 640 × 640, FasterGeo-YOLO achieves an mAP@0.5 of 0.567 and an mAP@0.5:0.95 of 0.286. After high-resolution refinement, the final model reaches a Precision of 0.632, Recall of 0.538, mAP@0.5 of 0.580, and mAP@0.5:0.95 of 0.293. Three-seed controlled ablation further confirms the reproducibility of the sequential design. The results demonstrate that FasterGeo-YOLO improves road damage detection while maintaining a lightweight and practically efficient model scale.

## 1. Introduction

As road service life increases, factors such as traffic loads, climate change, and material aging continuously aggravate pavement deterioration, leading to the gradual emergence of road damage such as cracks, alligator cracks, and potholes. Since early-stage road damage instances usually occupy small areas and have indistinct boundaries, delayed detection and maintenance may allow the damage to further expand under continuous traffic loads and environmental effects. This not only increases road maintenance costs and threatens traffic safety, but also affects driving comfort and the overall appearance of road infrastructure. Traditional manual inspection relies heavily on human experience and suffers from several limitations, including low efficiency, strong subjectivity, and difficulty in continuously covering large-scale road scenarios. Therefore, acquiring pavement conditions through image-capturing devices and automatically identifying road damage using intelligent detection algorithms has gradually become an important research direction in road inspection and maintenance management.

In this context, Maeda et al. collected road images using vehicle-mounted smartphones and applied deep neural networks to detect multiple types of road damage, demonstrating the feasibility of low-cost image acquisition and deep learning-based detection methods for road inspection [1]. Subsequently, Arya et al. further constructed a multi-national road damage dataset, extending road damage detection from single-region scenarios to complex cross-regional road environments [2]. The release of public datasets such as RDD2020 and RDD2022 has also provided a more representative experimental basis for detecting typical road damage, including longitudinal cracks, transverse cracks, alligator cracks, and potholes [3,4,5].

The road damage images in the RDD2022 dataset were collected from multiple countries and diverse road environments, with damage categories including longitudinal cracks, transverse cracks, alligator cracks, and potholes. Due to differences in road materials, viewing angles, illumination conditions, and pavement textures across regions, damages of the same category often exhibit considerable appearance variations in different scenarios. As a result, the model must not only identify damage categories, but also adapt to the instability of damage morphology and boundary characteristics under complex backgrounds.

Specifically, crack-like targets are usually slender, discontinuous, and small in scale, making their boundary information prone to loss during network downsampling. Alligator-crack regions have complex mesh-like topological structures, with noticeable texture variations inside the target regions.

Pothole targets, by contrast, are often characterized by weak boundaries, low contrast, and irregular region shapes.

Meanwhile, vehicle-mounted inspection and edge deployment scenarios require the model to perform real-time detection under constraints of limited parameters and computational cost. Therefore, lightweight models for road damage detection should not only have strong classification capability, but also maintain the ability to model small-scale, weak-boundary, and irregularly shaped targets under a limited computational budget.

To address these issues, this paper proposes FasterGeo-YOLO based on the lightweight YOLO26n detection framework. The network adopts a FasterNet-based backbone as an efficiency-oriented feature extractor and fuses multi-scale features through a YOLO-style neck to form P3, P4, and P5 detection scales. C2f_DCN deformable convolution modules are introduced at P3 and P4 to provide complementary geometric adaptation at different spatial resolutions rather than assigning individual damage types to a specific feature level. The enhanced P3 and P4 features, together with P5, are then used for classification and localization of the four road damage categories. After the network structure is determined, an 800 × 800 high-resolution refinement stage is applied to increase input spatial sampling density without changing the model structure or parameter count.

The name FasterGeo-YOLO reflects its two principal design components: “Faster” refers to the FasterNet-based lightweight feature extraction backbone, while “Geo” denotes geometric adaptive feature enhancement implemented by deformable convolution at the P3/P4 feature levels. The name describes the architectural composition rather than a blanket claim of superior frame rate across all hardware. Nevertheless, the revised manuscript now includes controlled profiling showing that the FasterNet redesign reduces forward latency by 15.32% and increases FPS by 18.09% relative to the matched-DFL control under the same 640 × 640 RTX 4090 D protocol.

The main contributions of this paper are summarized as follows:

(1) A task-oriented lightweight road damage detection framework, named FasterGeo-YOLO, is proposed. The framework combines an efficiency-oriented FasterNet backbone with selective geometric adaptive enhancement at the P3 and P4 feature levels. Controlled profiling directly verifies the practical inference-efficiency contribution of the FasterNet redesign, while the overall framework maintains a lightweight parameter scale.

(2) A P3/P4 dual-scale geometric adaptive enhancement structure is constructed to improve adaptation to irregular road damage morphology. Because fixed convolutional sampling is difficult to align with curved cracks, mesh-like structures, and fragmented pothole boundaries, C2f_DCN modules are introduced at P3 and P4. P3 retains finer spatial information and P4 provides complementary intermediate-resolution context; the revised manuscript therefore interprets them as complementary feature levels rather than assigning a particular damage category or object size exclusively to either level.

(3) A structure-aware high-resolution detail compensation strategy is proposed to further enhance the representation of weak-boundary and small-scale damages without changing the network structure or the number of parameters. After the network structure is determined, the best weights from the 640 × 640 training stage are loaded, and second-stage fine-tuning is performed at 800 × 800 resolution. This strategy increases the input spatial sampling density and compensates for local texture and boundary details that may be lost during downsampling. Compared with directly adding a P2 detection head, the proposed strategy avoids the additional computational overhead caused by high-resolution prediction layers and achieves a better accuracy–complexity trade-off in the experiments.

## 2. Related Work

### 2.1. Road Damage Detection Methods

Early road damage detection mainly relied on traditional image processing methods, such as edge detection, threshold segmentation, morphological processing, texture feature analysis, and hand-crafted feature-based classification. Such methods usually identify damaged regions based on gray-level changes, crack edges, or local texture differences, and have advantages such as simple implementation, low computational cost, and strong interpretability. However, road images are easily affected by illumination changes, shadow occlusion, lane markings, repair traces, and differences in pavement materials, making hand-crafted features difficult to adapt to variations in damage morphology under complex scenarios. Therefore, traditional methods have limited generalization capability in multi-class and multi-scenario road damage detection tasks.

With the development of convolutional neural networks and object detection techniques, deep learning-based road damage detection methods have gradually become the mainstream. Compared with traditional hand-crafted feature-based methods, deep learning models can automatically learn texture, boundary, and regional structural features of road damage from data, showing stronger adaptability in detecting multiple types of damages such as cracks, alligator cracks, and potholes. However, road damage targets differ significantly from common objects in natural scenes: cracks are usually slender and discontinuous, alligator cracks have complex mesh-like structures, and potholes often exhibit blurred boundaries and irregular shapes. Therefore, when general object detection models are directly applied to road damage detection, problems such as missed detection of small targets, unstable localization of weak boundaries, and false detection under complex backgrounds may still occur. Existing studies mostly focus on the overall application of detection frameworks or dataset generalization, while insufficient attention has been paid to how lightweight models can maintain fine-grained texture representation and irregular morphology modeling capability under low-parameter constraints.

### 2.2. Lightweight Object Detection Methods for Real-Time Deployment

For road damage detection tasks oriented toward vehicle-mounted inspection, mobile terminals, and edge computing platforms, models are required not only to achieve high detection accuracy but also to satisfy deployment requirements such as a small number of parameters, low computational cost, and fast inference. Therefore, real-time object detection frameworks and lightweight feature extraction networks have become important research directions. The YOLO series transforms object detection into an end-to-end regression problem and thus has obvious advantages in detection speed and engineering deployment [6]. Subsequently, methods such as YOLOv3, YOLOv4, and YOLOv7 continuously improved the backbone network, multi-scale detection structure, and training strategies, further enhancing real-time detection performance [7,8,9]. The Ultralytics YOLO framework provides a relatively complete pipeline for training, validation, and deployment, which also makes it highly applicable to practical scenarios such as road damage detection [10].

Meanwhile, lightweight network architectures have been widely used to reduce model complexity. MobileNetV2 reduces computational cost through depthwise separable convolution and inverted residual structures [11]; CSPNet alleviates redundant gradient computation through cross-stage feature partitioning and improves feature utilization efficiency [12]; EfficientDet combines compound scaling with efficient feature fusion to achieve a favorable balance between detection accuracy and computational cost [13]. These methods provide an effective foundation for real-time detection and edge deployment.

However, road damage targets differ significantly from common objects in natural scenes. Crack targets are usually slender and small in scale, alligator-crack targets have complex mesh-like structures, and pothole targets often exhibit blurred boundaries and irregular shapes. Although conventional lightweight detectors can satisfy real-time requirements, they tend to lose shallow boundary and local texture information during repeated downsampling and channel compression, resulting in insufficient representation of weak-boundary and small-scale damage targets. FasterNet reduces redundant computation and memory access through partial convolution, improving practical runtime efficiency while preserving spatial feature extraction capability, which makes it a suitable backbone choice for lightweight road damage detection [14]. Therefore, this paper adopts YOLO26n as the lightweight detection baseline and introduces a FasterNet backbone together with P3/P4 dual-scale geometric adaptive enhancement modules, aiming to improve the model’s ability to represent fine-grained textures and irregular morphologies of road damage under a low computational budget.

### 2.3. Multi-Scale Feature Fusion and Irregular Target Modeling Methods

Road damage targets exhibit significant scale variations. Longitudinal and transverse cracks usually appear as slender small targets, alligator-crack regions contain relatively complex local topological structures, and pothole targets may show large scale variations and irregular boundaries. Therefore, multi-scale feature representation is a key issue in road damage detection. FPN effectively improves multi-scale object detection by fusing high-level semantic information with low-level spatial information through a top-down pathway [15]. Based on this, PANet further enhances the transmission of low-level localization information to high-level semantic features [16]. EfficientDet achieves more efficient multi-scale fusion through a weighted bidirectional feature pyramid [13].

These multi-scale fusion methods can improve the detection performance for targets of different scales, but they still have certain limitations in lightweight road damage detection. On the one hand, adding a high-resolution detection layer, such as a P2 head, can provide more shallow details for small targets, but it also significantly increases the number of prediction points and computational cost. On the other hand, conventional multi-scale fusion mainly addresses information transmission among features at different scales, but it cannot directly solve the spatial misalignment between fixed convolutional sampling and irregular damage morphologies. Deformable convolution introduces learnable offsets into the sampling positions, allowing convolutional sampling points to be adjusted according to target shapes, which provides an effective solution for modeling irregular road damage targets [17,18]. Therefore, this paper focuses on introducing deformable convolution at the P3 and P4 scales, enabling the model to simultaneously enhance shallow boundary details and middle-level regional structural information.

In addition, to address the problems of small targets, weak textures, and complex backgrounds in road damage detection, existing studies have often introduced improvements from the perspectives of attention mechanisms, bounding box regression losses, and feature reconstruction strategies. For example, attention modules such as SE, CBAM, ECA, and SimAM can enhance responses in key regions through channel-wise or spatial reweighting [19,20,21,22]; loss functions such as NWD, GIoU, DIoU, and WIoU attempt to improve the localization of small targets and hard samples from the perspectives of bounding box distribution distance or dynamic focusing [23,24,25,26]; and dynamic upsampling methods such as CARAFE and DySample improve feature restoration quality through content-aware mechanisms [27,28].

Recent studies have also addressed object-detection degradation caused by adverse illumination and small target scales. Huang et al. proposed AMIEOD, which jointly optimizes multi-expert image enhancement and object detection to improve visual information utilization in low-illumination scenes [29]. For small-object detection, Jia et al. proposed EFAF-YOLO, which combines multi-scale feature enhancement, adaptive multi-scale feature fusion, and efficient multi-scale attention to strengthen small-object representation under complex backgrounds [30]. These studies demonstrate the effectiveness of illumination adaptation and multi-scale feature enhancement for challenging detection scenarios.

However, the above approaches primarily improve challenging-scene detection through feature weighting, illumination enhancement, loss constraints, multi-scale fusion, or feature reconstruction, and do not directly address the spatial misalignment between fixed convolutional sampling and highly irregular road damage morphology. Curved cracks, mesh-like alligator cracks, and fragmented pothole boundaries require the sampling locations themselves to adapt to geometric structure. Based on this consideration, this paper does not simply stack attention modules or extend detection heads, but instead focuses on geometry-adaptive sampling at the key P3/P4 scales.

## 3. Methods

### 3.1. Overall Architecture Overview

**Figure 1:**
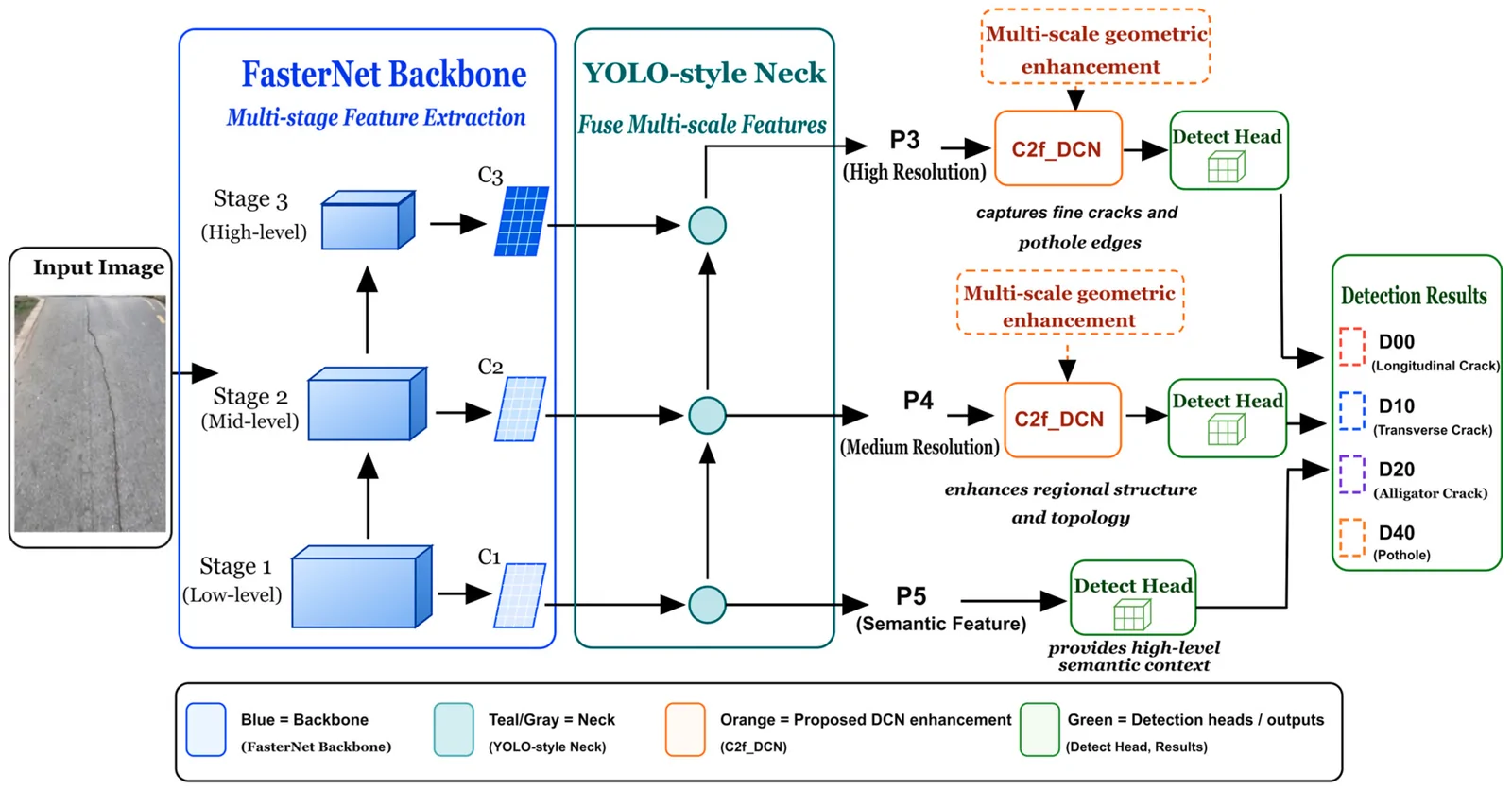
Overall Architecture of FasterGeo-YOLO.

The overall architecture of FasterGeo-YOLO is shown in Figure 1. The model follows a one-stage detection architecture and mainly consists of a lightweight FasterNet backbone, a YOLO-style multi-scale feature fusion neck, P3/P4 dual-scale geometric adaptive enhancement modules named C2f_DCN, and a three-scale detection head. The input road image is first processed by the FasterNet backbone to extract multi-level pavement texture and structural features, followed by multi-scale feature fusion through the neck. C2f_DCN modules are then introduced at the P3 and P4 feature levels to provide complementary deformable feature adaptation at different spatial resolutions. Finally, the enhanced features are fed into the P3, P4, and P5 three-scale detection heads to perform classification and bounding-box regression for D00, D10, D20, and D40 road damage.

Different from directly stacking attention modules or adding additional detection heads, FasterGeo-YOLO follows a selective geometric enhancement strategy. P3 retains relatively fine spatial information, whereas P4 provides complementary intermediate-resolution contextual representation. The revised interpretation does not assign a specific damage category or target size exclusively to either feature level. The high-level P5 feature is kept unchanged to preserve semantic stability and avoid unnecessary computational cost. Therefore, the proposed framework emphasizes complementary geometric adaptive modeling at selected feature levels rather than simply increasing model depth or feature-pyramid scale.

After the FasterNet + P3/P4-DCN structure is determined, this paper further adopts an 800 × 800 high-resolution fine-tuning strategy to enhance the detailed representation of weak-boundary and small-scale damages. This strategy improves the final detection performance by increasing the input spatial sampling density, without changing the network structure or the number of model parameters.

### 3.2. Problem Definition

Given a road image I ∈ *R*^(*H*×*W*×3)^, the objective of road damage detection is to predict a set of damage targets in the image:

(1) $$\hat{Y}={\left\{\left({\hat{b}}_{i},{\hat{c}}_{i},{\hat{s}}_{i}\right)\right\}}_{i=1}^{N}$$

where ${\hat{b}}_{i}=\left(x_{i},y_{i},w_{i},h_{i}\right)$ denotes the predicted bounding box, ${\hat{c}}_{i}$ ∈ {D00, D10, D20, D40} denotes the predicted category, ${\hat{s}}_{i}$ denotes the object confidence score, and N is the number of predicted targets. The objective of this paper is to improve the model’s detection capability for fine cracks, alligator-crack topological structures, and weak-boundary pothole regions under the constraints of a lightweight model.

### 3.3. FasterNet Lightweight Feature Extraction Backbone

Although the original YOLO26n backbone is lightweight, practical road damage detection requires a favorable balance between feature extraction capability and inference efficiency. Therefore, this paper adopts a FasterNet-based backbone redesign as an efficiency-oriented component. FasterNet is based on partial convolution, where only part of the channels participate in spatial feature modeling while the remaining channels preserve feature information. This design aims to reduce redundant memory access and improve practical execution efficiency while maintaining the feature representation required for road damage detection. The controlled profiling results in Section 4.6 directly evaluate this efficiency contribution.

*F* = ℬ*_FN_*(*X*) (2)

where ℬ*_FN_*(⋅) denotes the FasterNet feature extraction module, X denotes the input feature, and F denotes the output feature. The multi-scale features produced by the FasterNet backbone are used as the basic feature representations for the subsequent P3/P4 dual-scale geometric adaptive enhancement.

### 3.4. Dual-Scale Geometric Adaptive Enhancement Module

Standard convolution adopts a fixed sampling grid, which is efficient for regular targets but difficult to adapt to the irregular morphology of road damage targets. Cracks may appear as curved, fractured, and slender distributions; alligator cracks usually exhibit mesh-like topological structures; and pothole boundaries may be fragmented and mixed with the pavement background. Fixed convolutional sampling points are difficult to fully align with the actual contours of these damages, thereby affecting the model’s representation capability for weak boundaries and irregular regions.

For standard convolution, its output can be expressed as:

(3) $$y(p_{0})=\sum_{p_{n}\in R}w(p_{n})\cdot x(p_{0}+p_{n})$$

where *R* denotes the regular convolutional sampling grid, *p*_0_ is the output position, *p_n_* is the fixed offset position, *w*(*p_n_*) represents the convolution weight, and *x*(⋅) denotes the input feature map. Since *p_n_* is fixed, standard convolution cannot adaptively adjust its sampling region according to the actual morphology of road damage.

Deformable convolution learns spatial offsets through an offset prediction branch:

(4) $$\Delta P=f_{\mathrm{offset}}(X),\;\Delta P={\left\{\Delta p_{n}\right\}}_{n=1}^{\left|R\right|}$$

where *f*_offset_(·) denotes the offset prediction function, Δ*P* denotes the set of predicted offsets, and Δ*p_n_* denotes the learnable spatial offset corresponding to the sampling point indexed by *n*. For a 3 × 3 convolution kernel, offsets in both the horizontal and vertical directions need to be predicted for the 9 sampling points; therefore, the number of offset channels is usually 2∣R∣. After introducing the offsets, the output of deformable convolution can be expressed as:

(5) $$y(p_{0})=\sum_{p_{n}\in R}w(p_{n})\cdot x(p_{0}+p_{n}+\Delta p_{n})$$

Since *p*_0_ + *p_n_*+Δ*p_n_* is usually not an integer coordinate, the feature value at the corresponding position is obtained by bilinear interpolation in practical computation:

(6) $$x(p)=\sum_{q\in N(p)}G(q,p)\cdot x(q)$$

where *p* = *p*_0_ + *p_n_* + Δ*p_n_*, q∈N(p) denotes a neighboring integer grid position around *p*, and *G*(*q*,*p*) represents the bilinear interpolation weight between *q* and *p*. Through the above adaptive sampling process, the convolutional sampling points can better fit curved cracks, mesh-like alligator-crack structures, and irregular pothole boundaries.

Let the P3, P4, and P5 features output by the neck be denoted as the corresponding multi-scale feature maps. This paper introduces C2f_DCN modules into P3 and P4 to construct a dual-scale geometric adaptive enhancement structure. P3 retains a relatively high spatial resolution and therefore provides finer local spatial information, whereas P4 supplies complementary intermediate-resolution contextual and regional representation. These properties motivate the use of both feature levels, but they are not interpreted as a strict one-to-one assignment between a feature level and a particular road damage category or target size.

(7) $${\tilde{F}}_{3}=C2f\_\mathrm{DCN}(F_{3}),\;{\tilde{F}}_{4}=C2f\_\mathrm{DCN}(F_{4})$$

(8) $$\hat{Y}=\mathrm{Detect}({\tilde{F}}_{3},{\tilde{F}}_{4},F_{5})$$

The enhanced P3 and P4 features are used together with the original P5 semantic feature in the detection head. In this design, P3 and P4 provide complementary geometric adaptation at different spatial resolutions, while P5 preserves high-level semantics. Compared with applying DCN to all feature scales, selectively enhancing P3 and P4 concentrates adaptive sampling on two informative feature levels while avoiding unnecessary computational overhead.

**Figure 2:**
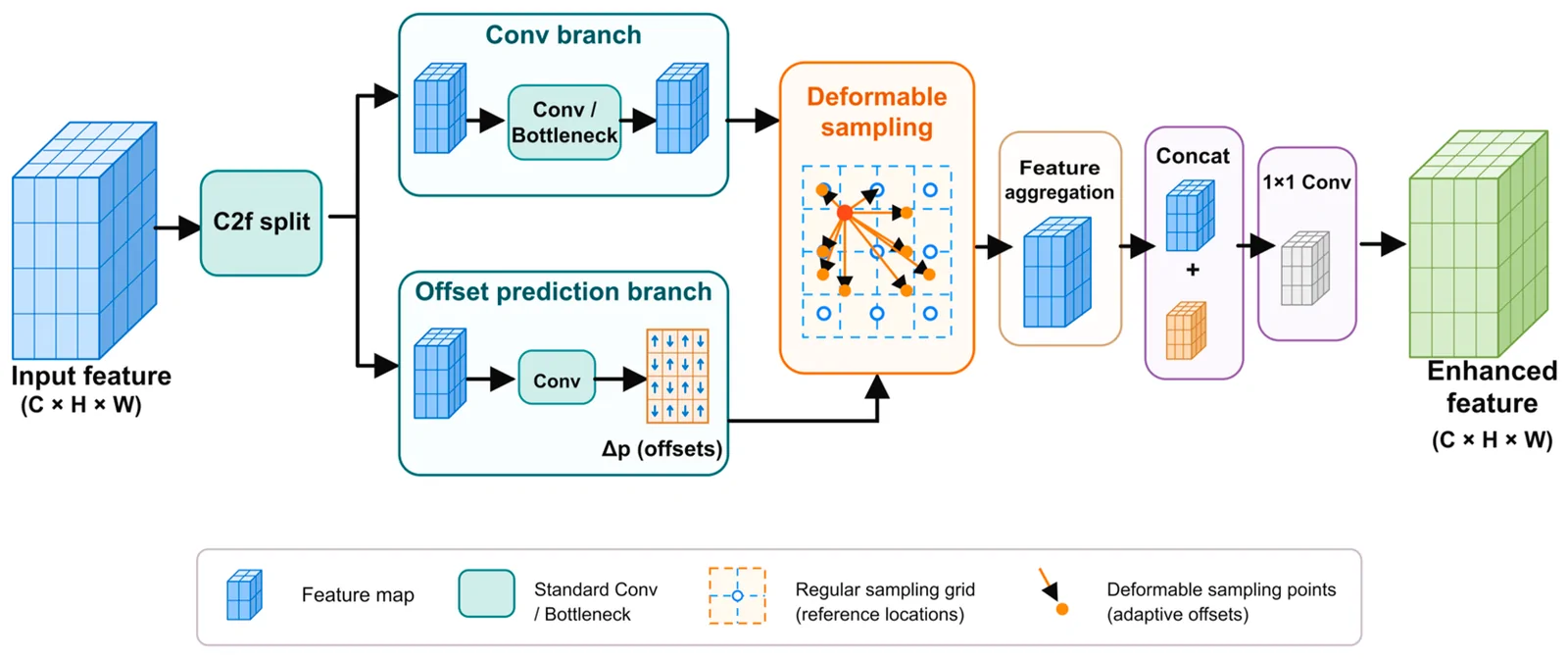
Structure of the C2f_DCN Module.

As shown in Figure 2, the C2f_DCN module first divides the input features along the channel dimension, so as to preserve the lightweight feature representation capability of the C2f structure. During feature transformation, part of the features are processed by standard convolution or Bottleneck structures to extract local texture information, while an offset prediction branch generates learnable spatial offsets to guide the convolutional sampling positions to adapt to the morphology of road damage targets. Then, deformable convolution performs geometric adaptive sampling over local regions according to the predicted offsets, and enhanced geometric features are obtained through feature aggregation. Finally, the geometrically enhanced features are concatenated with the preserved branch features, followed by a 1 × 1 convolution for channel fusion to produce the final output features. This process maintains the lightweight advantage of the C2f module while improving the adaptability of convolutional sampling to curved cracks, mesh-like alligator cracks, and irregular pothole boundaries.

### 3.5. YOLO-Style Multi-Scale Detection Head

This paper retains a YOLO-style three-scale prediction structure that takes P3, P4, and P5 features as inputs. However, the regression formulation is intentionally modified relative to the exact YOLO26n baseline. The original YOLO26n baseline uses its native DFL-free formulation (reg_max = 1 and E2E loss), whereas FasterGeo-YOLO uses a distributional regression head (reg_max = 16) and reintroduces Distribution Focal Loss (DFL). This task-oriented modification is intended to strengthen bounding-box distribution regression for road damages with irregular shapes and weak boundaries. No additional P2 detection layer is introduced, thereby maintaining a favorable balance between detailed representation and model complexity.

### 3.6. Structure-Aware High-Resolution Detail Compensation Optimization Method

After determining the FasterNet + P3/P4-DCN structure, this paper further introduces a structure-aware high-resolution detail compensation optimization method to improve the model’s fine-detail perception capability for small-scale damages, weak-boundary regions, and low-contrast targets. Here, “structure-aware” does not mean introducing an additional structural branch. Instead, it indicates that high-resolution fine-tuning is performed after the FasterNet + P3/P4-DCN geometric enhancement structure has been determined, so that the additional spatial details provided by the higher input resolution can be further utilized by the dual-scale geometric enhancement modules. This method adopts a two-stage training process.

In the first stage, standard training is performed at an input resolution of 640 × 640 to obtain the base model weights:

(9) $$\theta_{640}^{*}=\mathrm{argmi}n_{\theta }L_{\det}(I_{640},Y;\theta )$$

**Figure 3:**
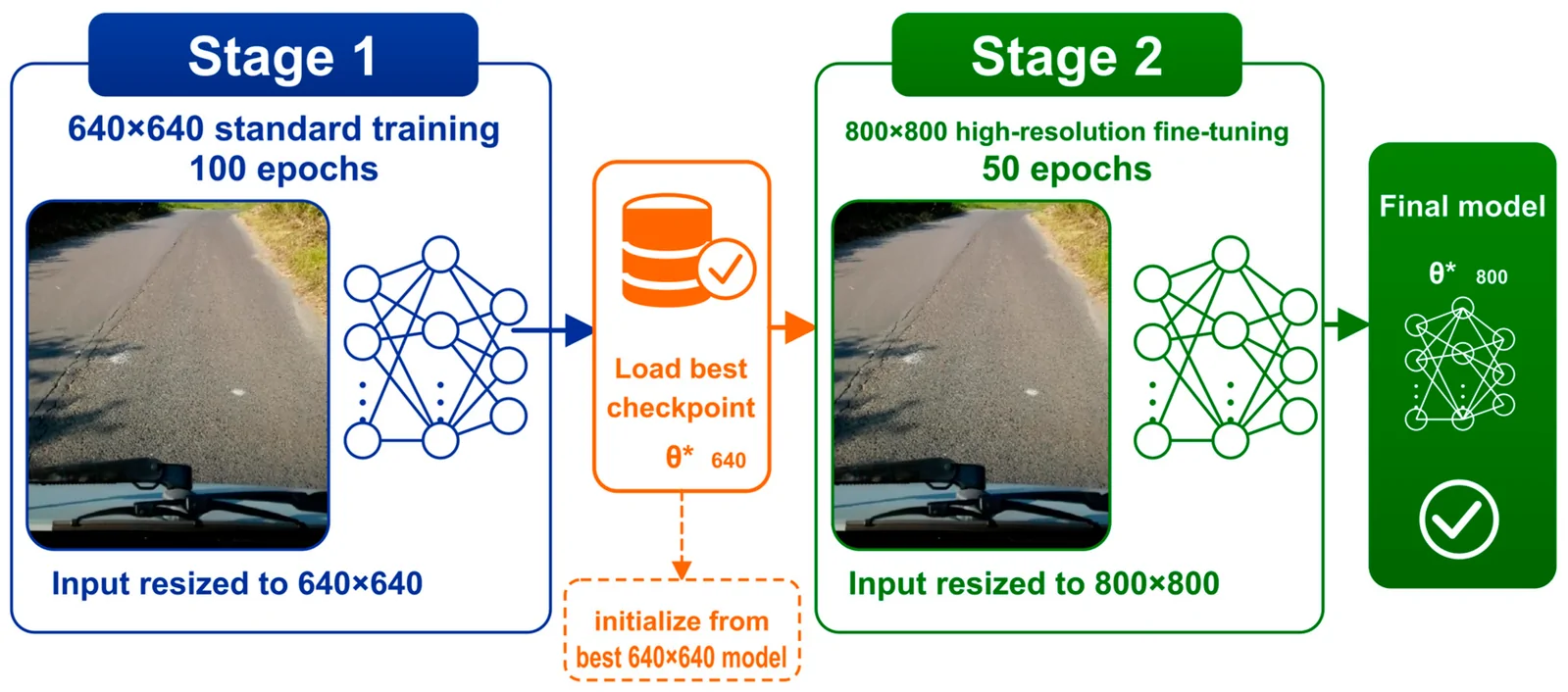
Two-stage High-resolution Detail-compensation Optimization Process.

In the second stage, the best weights obtained from the first stage are loaded, and fine-tuning is continued at an input resolution of 800 × 800:

(10) $$\theta^{\left(0\right)}=\theta_{640}^{*},\;\theta_{800}^{*}=\mathrm{argmi}n_{\theta }L_{\det}(I_{800},Y;\theta )$$

where $\theta_{640}^{*}$ denotes the optimal weights obtained from training at the 640 resolution, and $\theta_{800}^{*}$ denotes the final weights after high-resolution detail-compensation optimization. This method does not change the network structure or the number of parameters; instead, it increases the spatial sampling density by improving the input image resolution, thereby compensating for local texture and weak-boundary details that may be lost during downsampling. The overall two-stage high-resolution detail-compensation process is illustrated in Figure 3.

### 3.7. Training Objective Function

The loss formulation in this section refers specifically to FasterGeo-YOLO rather than to the exact YOLO26n baseline. The exact YOLO26n control remains DFL-free, while FasterGeo-YOLO intentionally reintroduces DFL as part of its modified regression formulation. A matched-DFL intermediate control is included in Section 4.3 to isolate the contribution of this change.

For FasterGeo-YOLO, a YOLO-style detection loss is used to optimize the model in an end-to-end manner. Given the training samples $D={\left\{\left(I_{i},Y_{i}\right)\right\}}_{i=1}^{M}$, where denotes the *i* training image, *Y_i_* denotes its corresponding annotation, and *M* denotes the number of training samples, the optimization objective of the model parameters is formulated as:

(11) $$\theta^{*}=\mathrm{argmi}n_{\theta }\frac{1}{M}\sum_{i=1}^{M}L_{\det}(I_{i},Y_{i};\theta )$$

where ℒ*_det_* denotes the detection loss, which consists of the bounding box regression loss, classification loss, and Distribution Focal Loss:

ℒ*_det_* = *λ_box_*ℒ*_box_* + *λ_cls_*ℒ*_cls_* + *λ_dfl_*ℒ*_dfl_* (12)

where ℒ*_box_* is used to constrain the localization difference between the predicted boxes and the ground-truth boxes, ℒ*_cls_* is used to optimize the damage category prediction, and ℒ*_dfl_* is used to improve the accuracy of bounding box distribution regression. According to the experimental settings, this paper sets *λ_box_* = 7.5, *λ_cls_* = 0.5, *λ_dfl_* = 1.5.

## 4. Experimental Results and Analysis

### 4.1. Experimental Setup

The experiments are conducted on the cleaned RDD2022 road damage dataset using a fixed 7:3 training/validation split. The retained dataset contains independent road damage images rather than temporally consecutive frames or continuous route/video sequences. Therefore, the same-route or adjacent-frame leakage mechanism that can occur in frame-based datasets does not arise under the present data organization. On this basis, no additional untouched test subset was separately constructed. The training set contains 16,636 images, while the validation set contains 7131 images with 16,479 target instances. The detection categories are D00, D10, D20, and D40, corresponding to longitudinal cracks, transverse cracks, alligator cracks, and potholes. All annotations are cleaned and checked for category consistency, and the same fixed split is used across baseline, ablation, and controlled experiments.

The YOLO-series models, road damage improved models, and FasterGeo-YOLO do not load external pretrained weights; the 800 × 800 stage loads only the best weights from the corresponding 640 × 640 model for high-resolution refinement. Faster R-CNN R50-FPN and RetinaNet R50-FPN are initialized with official TorchVision COCO-pretrained weights, while RT-DETR-L uses Ultralytics official COCO-pretrained weights and is included as a representative detection-paradigm reference. RDD2022 contains images collected from multiple countries and provides substantial visual diversity in pavement materials, viewpoints, illumination, and background textures. However, the present fixed within-dataset split should not be interpreted as a strict country-level geographic generalization test. Country-wise, leave-one-country-out, and independent external-dataset evaluations are reserved for future work.

The evaluation metrics include Precision, Recall, mAP@0.5, mAP@0.5:0.95, the number of parameters, GFLOPs@640, and single-image inference time. Specifically, Precision measures the proportion of correctly detected targets among the model’s predictions, while Recall measures the proportion of ground-truth damage targets that are successfully detected. mAP@0.5 reflects the overall detection performance at an IoU threshold of 0.5, whereas mAP@0.5:0.95 better reflects the localization quality of the model under different IoU thresholds. The dataset characteristics and experimental settings are summarized in Table 1.

### 4.2. Multi-Model Comparative Experiment

To evaluate FasterGeo-YOLO against representative detectors under a clearer comparison protocol, this paper includes Faster R-CNN R50-FPN, RetinaNet R50-FPN, RT-DETR-L, YOLOv5nu, YOLOv8n, YOLO11n, YOLO26n [10,31,32,33], OBC-YOLOv8, and Improved YOLOv8s [34,35]. To avoid confounding architectural differences with input resolution, all models in the main baseline comparison of Table 2 are reported at 640 × 640 input resolution. The YOLO-series models, road damage improved models, and FasterGeo-YOLO are trained without external pretrained weights. COCO-pretrained Faster R-CNN, RetinaNet, and RT-DETR-L are retained as representative references for different detection paradigms rather than as strictly identical-pretraining comparisons. The subsequent 800 × 800 stage is discussed separately as a high-resolution refinement strategy and is not used to establish the architectural advantage in Table 2.

It should be noted that the original implementations of OBC-YOLOv8 and Improved YOLOv8s are based on older versions of the Ultralytics framework. Therefore, the SGD optimizer supported by their original code frameworks is used in the reproduction experiments. For Improved YOLOv8s, the complete DyHead version cannot be used because the ModulatedDeformConv2d operator is unavailable in the current environment; consequently, this paper reports the result of its C2f-Faster-EMA + SlimNeck variant.

As shown in Table 2, all main comparison results use 640 × 640 input. YOLO11n achieves an mAP@0.5 of 0.546 and an mAP@0.5:0.95 of 0.275, while the exact YOLO26n baseline achieves 0.522 and 0.258. Under the same 640 × 640 resolution, FasterGeo-YOLO reaches a Precision of 0.612, Recall of 0.540, mAP@0.5 of 0.567, and mAP@0.5:0.95 of 0.286. Thus, the baseline comparison reflects the advantage of the proposed structure without using the later 800 × 800 refinement result. The high-resolution refinement stage is analyzed separately in Section 4.4.

### 4.3. Ablation Experiments

#### 4.3.1. Controlled Sequential Ablation and Structural Evolution

**Figure 4:**
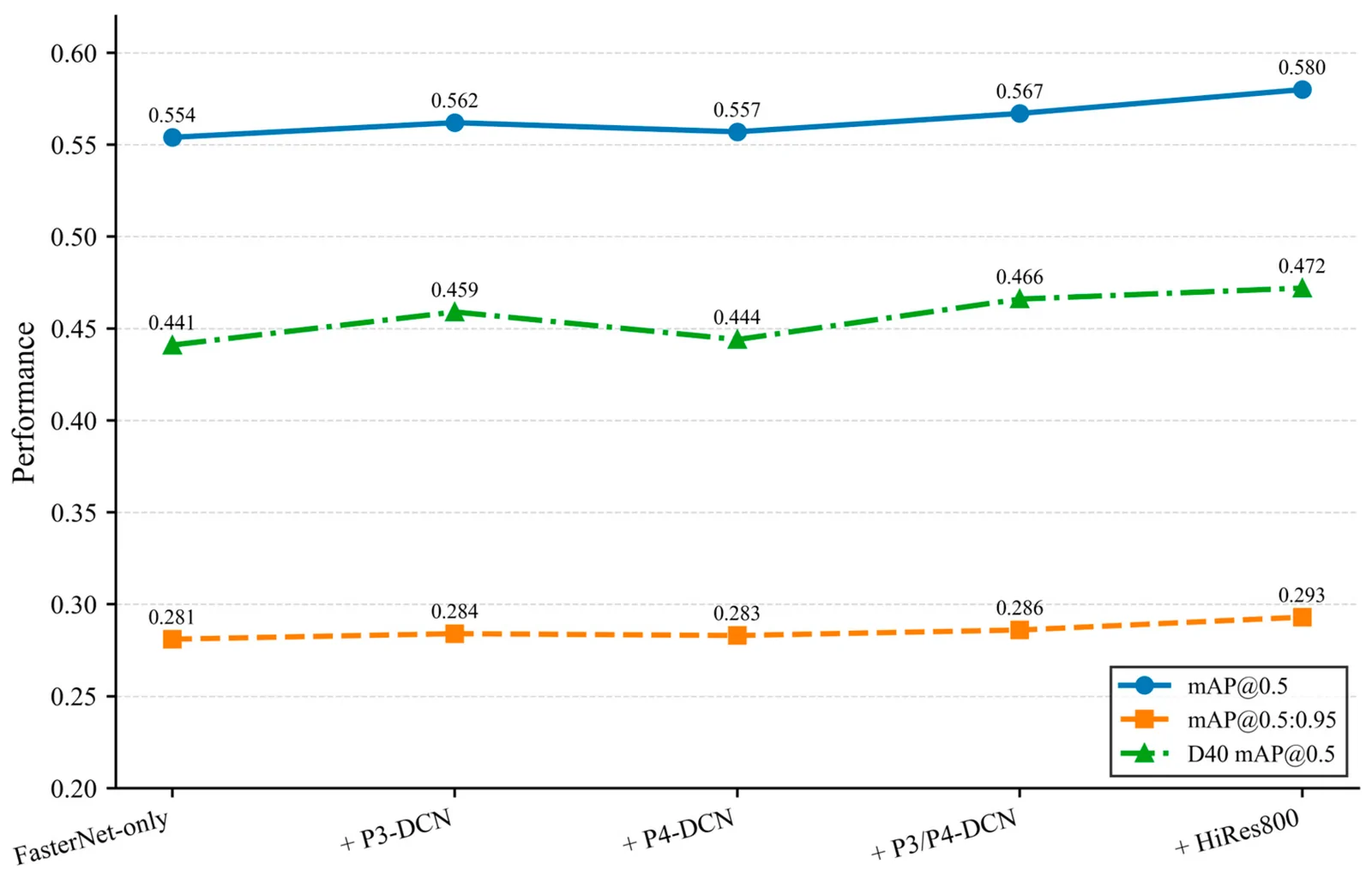
Changes in mAP metrics during structural evolution.

To separate the contributions of detection formulation, backbone redesign, deformable enhancement, and high-resolution refinement, we performed a controlled sequential ablation using three training seeds (0, 1, and 2). Step0–Step3 were independently trained from scratch for 100 epochs at 640 × 640 using the same fixed split, optimizer, and hyperparameters. Step4 loads the same-seed Step3 best checkpoint and performs an additional 50-epoch refinement at 800 × 800. Table 3 reports mean ± sample standard deviation across the three seeds. Figure 4 is retained as a visual overview of the structural evolution, while the controlled statistics in Table 3 are used for quantitative attribution.

#### 4.3.2. Analysis of DCN Insertion Positions

**Figure 5:**
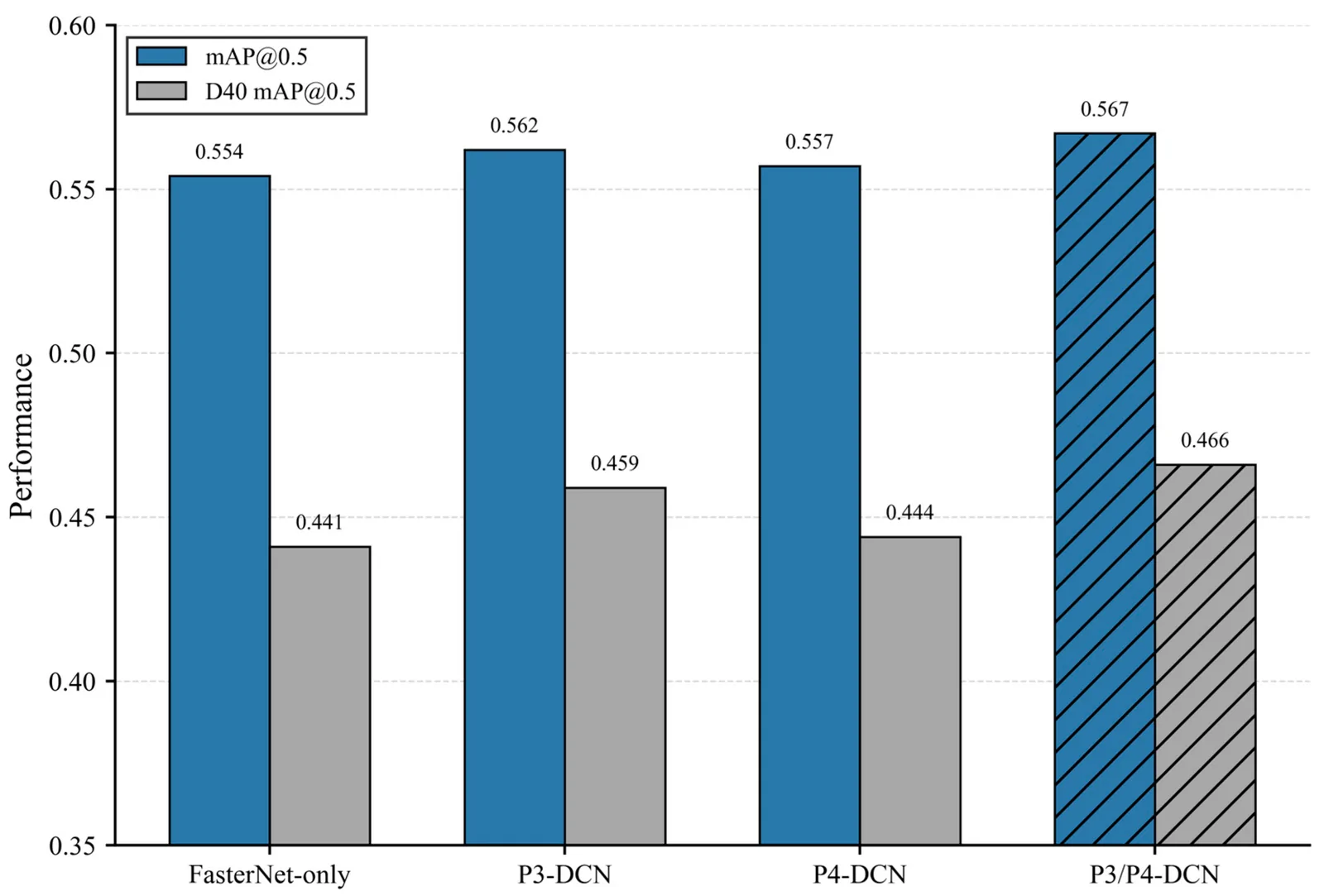
Ablation Results of DCN Insertion Positions.

From the perspective of DCN insertion positions, P3 provides a higher-resolution feature representation, whereas P4 contributes complementary intermediate-resolution contextual information. The revised manuscript therefore does not assign fine cracks exclusively to P3 or larger damage topology exclusively to P4. Instead, the insertion-position experiment evaluates whether the two feature levels provide complementary deformable adaptation. The results are illustrated in Figure 5 and summarized in Table 4.

#### 4.3.3. Quantitative Multi-Seed and Insertion-Position Analysis

The controlled results in Table 3 show a progressive and reproducible construction process. The exact DFL-free YOLO26n control obtains 0.5225 ± 0.0043 mAP@0.5 and 0.2598 ± 0.0014 mAP@0.5:0.95. Introducing the matched DFL formulation increases these metrics to 0.5496 ± 0.0026 and 0.2761 ± 0.0009, respectively. The FasterNet-based redesign then preserves the accuracy level while providing a measurable practical speed benefit, as further quantified in Section 4.6.

Starting from the FasterNet configuration, P3-DCN increases the three-seed mean mAP@0.5 from 0.5515 ± 0.0015 to 0.5539 ± 0.0029 and mAP@0.5:0.95 from 0.2767 ± 0.0008 to 0.2788 ± 0.0021. Adding P4-DCN on top of P3 further raises the metrics to 0.5613 ± 0.0012 and 0.2831 ± 0.0004. The subsequent 800 × 800 refinement reaches 0.5750 ± 0.0026 and 0.2874 ± 0.0020. Because Step4 changes both resolution and training budget, its gain is interpreted as a high-resolution refinement effect rather than as a pure architectural contribution.

The separate insertion-position experiment is retained to distinguish P3-only, P4-only, and joint P3/P4 configurations. FasterNet-only, P3-only, P4-only, and P3/P4 configurations obtain mAP@0.5 values of 0.554, 0.562, 0.557, and 0.567, respectively. The joint configuration performs best overall, supporting complementary multi-scale adaptation rather than a rigid scale-to-damage assignment.

The class-wise three-seed analysis also supports a complementary rather than category-exclusive interpretation. Mean mAP@0.5 values for Step1/Step2/Step3 are D00: 0.5527/0.5573/0.5620, D10: 0.5490/0.5467/0.5583, D20: 0.6637/0.6650/0.6703, and D40: 0.4403/0.4473/0.4550. The gains are distributed across multiple categories, so the revised manuscript does not claim a strict one-to-one correspondence between P3 or P4 and a specific damage class or target size.

### 4.4. Analysis of Alternative Schemes and Optimization Strategies

To further verify the rationality of the proposed structural design, this paper compares several 640 × 640 alternatives, including loss-function replacement, dynamic upsampling, and a high-resolution P2 detection head. These structural alternatives are summarized in Table 5. The 800 × 800 high-resolution refinement is intentionally excluded from this table and discussed separately in the text so that architectural alternatives and resolution/training-budget effects are not mixed.

As shown in the results, although bounding box regression losses such as NWD and WIoU can adjust localization constraints to a certain extent, their overall mAP@0.5 and mAP@0.5:0.95 do not surpass those of the main P3/P4-DCN structure, and no clear improvement is observed for the D40 pothole category. DySample achieves a slight improvement in mAP@0.5:0.95, but its D40 mAP@0.5 remains lower than that of the main structure, indicating that dynamic upsampling provides limited improvement for weak-boundary potholes.

The P2 head introduces more high-resolution prediction points and raises computational cost to 10.4 GFLOPs@640 without improving D40 mAP@0.5 over the main P3/P4 structure. In contrast, the separate HiRes800 refinement does not change the network structure or parameter count; it fine-tunes the 640 × 640 model at 800 × 800 and reaches an mAP@0.5 of 0.580 and an mAP@0.5:0.95 of 0.293 in the representative final run. Accordingly, the high-resolution result is treated as a training/refinement strategy rather than as part of the equal-resolution structural comparison.

The effect of the high-resolution refinement strategy is evaluated separately using a resolution-only YOLO26n control. YOLO26n improves from P = 0.590, R = 0.504, mAP@0.5 = 0.522, and mAP@0.5:0.95 = 0.258 at 640 × 640 to 0.628, 0.539, 0.569, and 0.284 after the 800 × 800 refinement. FasterGeo-YOLO similarly improves from the 640 × 640 main-structure result to 0.580 mAP@0.5 and 0.293 mAP@0.5:0.95 after high-resolution refinement. This demonstrates that the 800 × 800 stage is an effective training strategy for both the baseline and the proposed model rather than an advantage exclusive to FasterGeo-YOLO. Under the same 800 × 800 setting, FasterGeo-YOLO still exceeds YOLO26n + HiRes800 by 0.011 mAP@0.5 and 0.009 mAP@0.5:0.95.

### 4.5. Class-Wise Result Analysis

To further analyze the detection capability of the final FasterGeo-YOLO model for different road damage categories, Table 6 reports the class-wise results of the representative final 800 × 800 model for D00, D10, D20, and D40. These results characterize category-level behavior of the final detector and are distinguished from the controlled P3/P4 class-wise ablation reported in Section 4.3.3.

As shown in the results, FasterGeo-YOLO achieves the best detection performance on the D20 alligator-crack category, with Precision, Recall, mAP@0.5, and mAP@0.5:0.95 reaching 0.682, 0.616, 0.672, and 0.354, respectively. Alligator-crack regions usually contain relatively distinct mesh-like textures and locally connected structures. Although their morphology is complex, their regional characteristics are relatively prominent. After introducing P3/P4 dual-scale geometric adaptive enhancement modules, the model can better adapt to the irregular morphology of alligator-crack targets and jointly represent their local textures and regional structures, thereby achieving higher detection accuracy for this category.

For the D00 longitudinal crack and D10 transverse crack categories, the final model also achieves relatively stable detection results. Specifically, the mAP@0.5 and mAP@0.5:0.95 of D00 are 0.596 and 0.329, while those of D10 are 0.582 and 0.285. Crack-like targets are often slender and discontinuous, making local details vulnerable to repeated downsampling. The complementary P3/P4 deformable adaptation, together with high-resolution refinement, improves the preservation and use of spatial detail; however, these observations are not interpreted as evidence that a single feature level exclusively handles a particular crack type or object size.

In contrast, D40 potholes remain the most challenging category. The Precision, Recall, mAP@0.5, and mAP@0.5:0.95 of this category are 0.586, 0.425, 0.472, and 0.206, respectively, which are overall lower than those of the other three damage categories. The main reason is that pothole targets often exhibit weak boundaries, low contrast, and irregular regional shapes, and they are easily affected by shadows, pavement repair traces, material variations, and complex background textures. Although high-resolution detail-compensation optimization can increase the spatial sampling density of input images and improve the model’s perception of local textures and edge regions of potholes, rectangular bounding boxes still have difficulty accurately describing the fragmented and irregular true contours of potholes under high IoU thresholds. Therefore, the mAP@0.5:0.95 of D40 remains relatively low.

Overall, FasterGeo-YOLO achieves effective detection performance across all four types of road damage. It shows better adaptability to alligator cracks and crack-like targets, while potholes remain the main challenge limiting further model improvement. This also indicates that P3/P4 dual-scale geometric adaptive enhancement and high-resolution detail compensation optimization can effectively improve the feature representation of road damage. However, for targets such as weak-boundary and irregular potholes, it is still necessary to further improve localization accuracy by incorporating instance segmentation, boundary point modeling, or more fine-grained region representation methods in future work.

### 4.6. Efficiency Analysis

The computational complexity and runtime efficiency were re-profiled using a unified protocol. The unfused FasterGeo-YOLO architecture contains 2,761,616 parameters (2.762 M). At 640 × 640 input, it requires 7.059 GFLOPs, whereas the final 800 × 800 setting requires 11.030 GFLOPs. Runtime was measured on an NVIDIA RTX 4090 D using FP32 precision and batch size 1. After 100 warm-up iterations, 500 CUDA-synchronized network-forward passes were timed. The final 800 × 800 model achieves 8.860 ± 1.763 ms per image, corresponding to 112.86 FPS, with 109.11 MB peak allocated GPU memory. These values replace the previous mixed-resolution complexity and latency figures.

To directly assess the efficiency contribution of the FasterNet redesign, we additionally profiled the matched-DFL YOLO26n control and FasterNet configuration at 640 × 640 under the same protocol. StepH requires 2.694 M parameters, 6.599 GFLOPs, 8.489 ± 0.622 ms, and 118.23 ± 9.02 FPS, whereas Step1 FasterNet requires 2.642 M parameters, 6.656 GFLOPs, 7.189 ± 0.546 ms, and 139.62 ± 10.18 FPS. Thus, FasterNet reduces parameter count by 1.92% and forward latency by 15.32% while increasing FPS by 18.09%, despite a small 0.87% increase in nominal GFLOPs. This result indicates that practical GPU execution efficiency is not fully characterized by FLOPs alone and supports the role of FasterNet as an efficiency-oriented backbone redesign rather than as a large standalone accuracy-gain module.

### 4.7. Qualitative Analysis and Visualization

**Figure 6:**
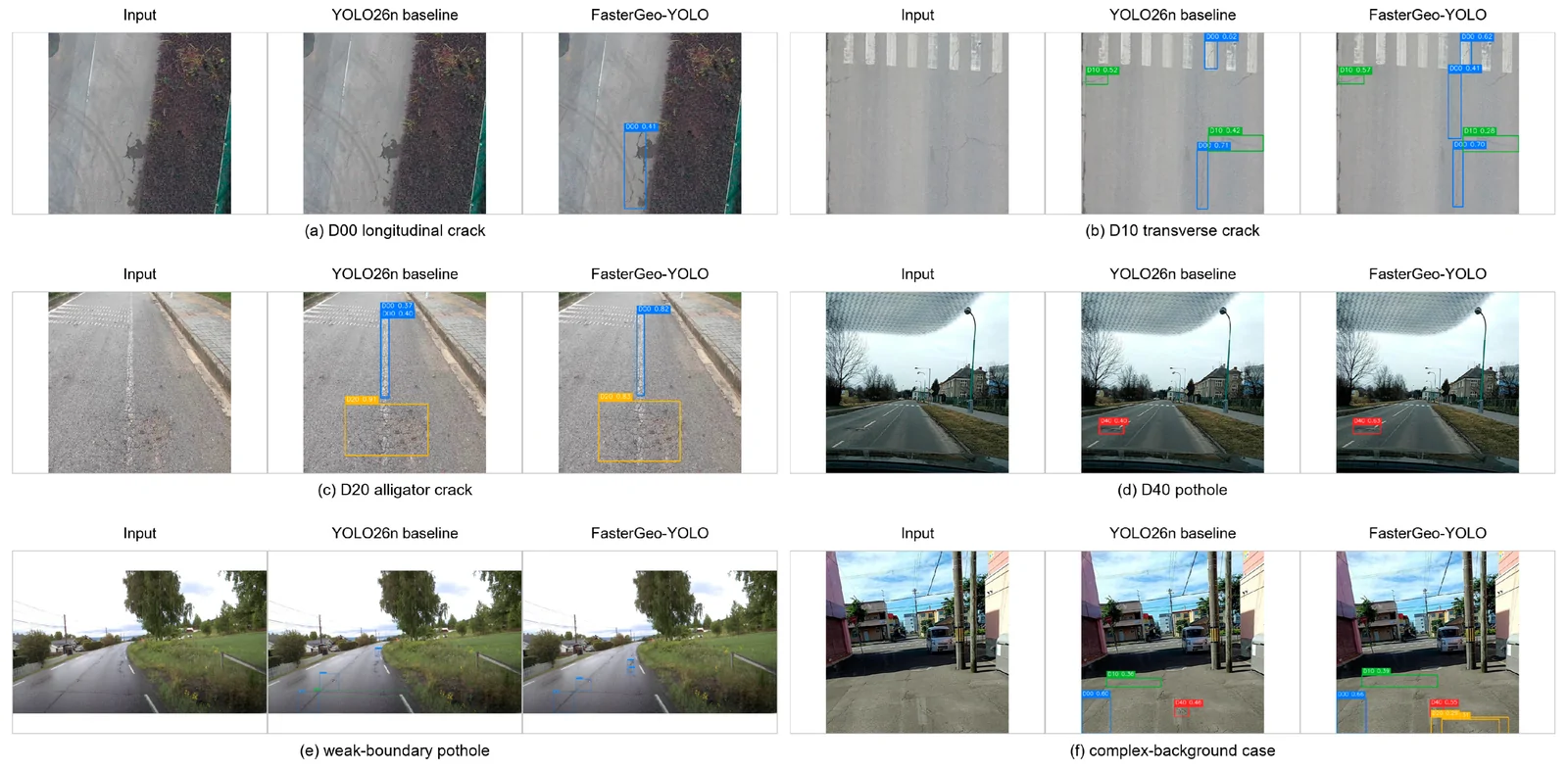
Qualitative Detection Result Comparison between the YOLO26n Baseline and FasterGeo-YOLO.

In addition to quantitative metrics, qualitative visualization can more intuitively demonstrate whether the model focuses on appropriate damage regions. As shown in Figure 6, this paper selects representative samples, including longitudinal cracks, transverse cracks, alligator cracks, potholes, weak-boundary potholes, and complex backgrounds, to compare the detection results of the YOLO26n baseline and FasterGeo-YOLO. The results show that the baseline is prone to missed detections, low confidence scores, or unstable localization in scenarios involving slender cracks and weak-boundary damages. In contrast, FasterGeo-YOLO provides more stable detection results across multiple typical damage categories and challenging scenarios.

**Figure 7:**
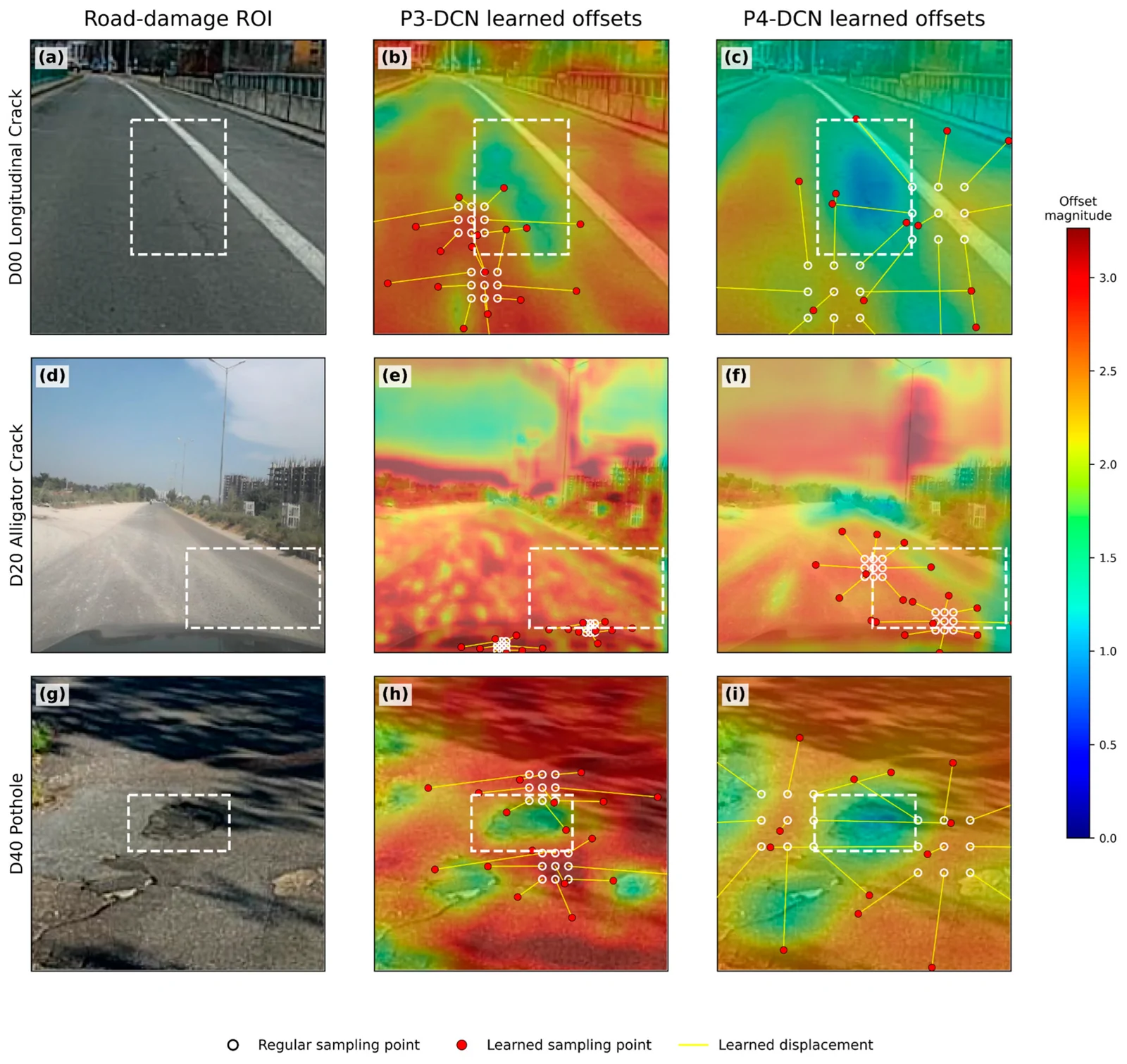
Visualization of learned deformable offsets at the P3 and P4 feature levels for representative road damage regions. (**a**) D00 longitudinal crack ROI; (**b**) P3-DCN learned offsets for D00; (**c**) P4-DCN learned offsets for D00; (**d**) D20 alligator crack ROI; (**e**) P3-DCN learned offsets for D20; (**f**) P4-DCN learned offsets for D20; (**g**) D40 pothole ROI; (**h**) P3-DCN learned offsets for D40; (**i**) P4-DCN learned offsets for D40. White dashed rectangles indicate the selected regions of interest (ROIs). White hollow circles denote regular sampling points, red points denote learned sampling points, yellow lines indicate learned displacements, and the background heatmap represents offset magnitude.

To directly examine the behavior of deformable sampling, Figure 7 visualizes learned offsets at the P3 and P4 feature levels for representative D00, D20, and D40 road damage regions. White hollow circles denote regular sampling locations, red points denote learned sampling locations, yellow line segments indicate learned displacements, and the background heatmap represents offset magnitude. Both feature levels exhibit spatially non-uniform adaptive sampling around damage structures. P3 shows finer local displacement patterns because of its higher spatial resolution, whereas P4 provides complementary coarser feature-level adaptation. These visualizations are interpreted as qualitative evidence of adaptive geometric sampling rather than as a strict assignment of individual damage categories or target sizes to a particular feature level.

**Figure 8:**
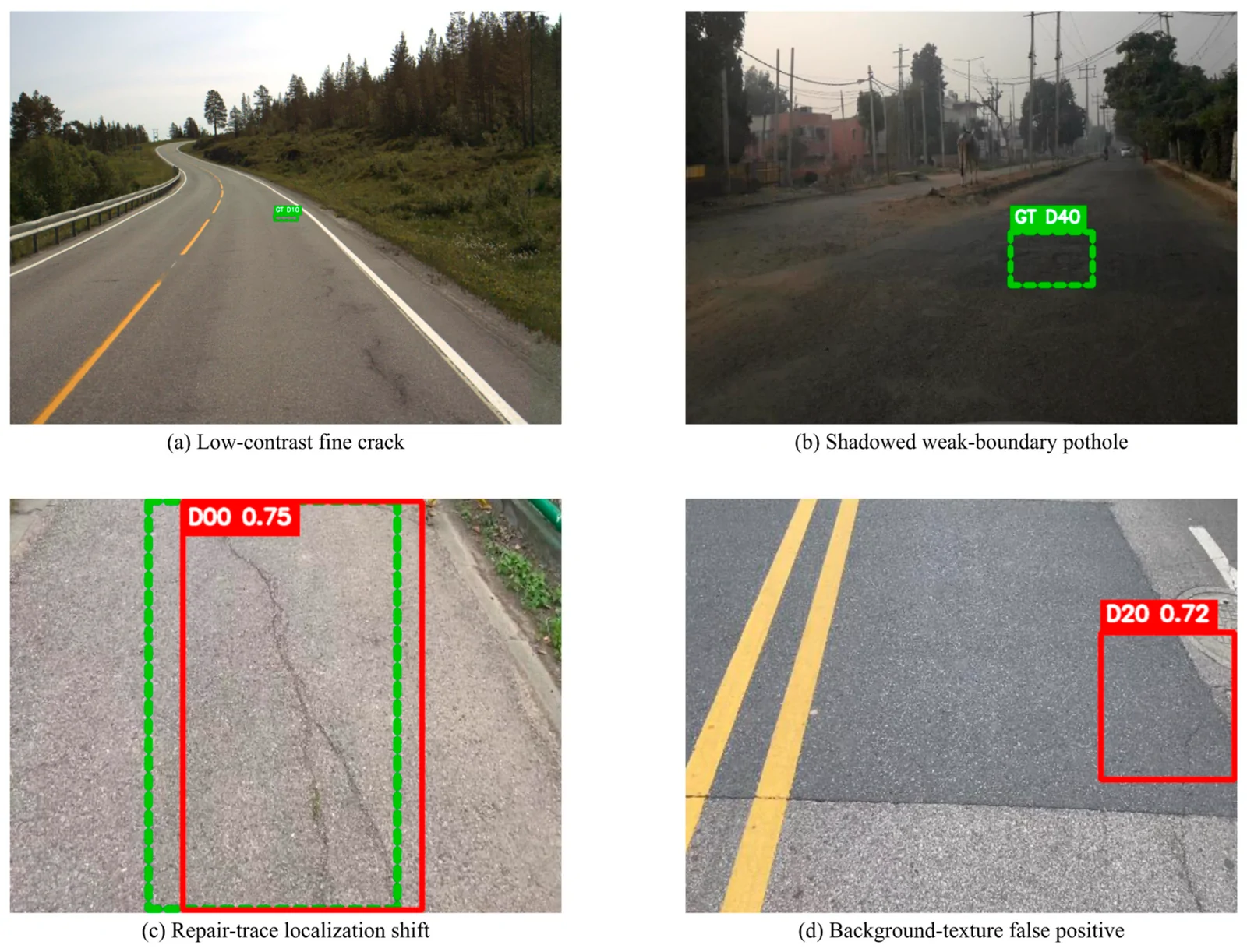
Representative failure cases of FasterGeo-YOLO on RDD2022. (**a**) Missed detection of a low-contrast fine crack; (**b**) missed detection of a shadowed weak-boundary pothole; (**c**) localization shift caused by pavement repair traces; and (**d**) false positive induced by confusing background texture. Green dashed boxes denote ground-truth annotations, whereas red solid boxes denote model predictions.

Representative failure cases of FasterGeo-YOLO are shown in Figure 8. Low-contrast fine cracks may be missed when their visual response is only slightly different from the surrounding pavement, while weak-boundary potholes can become difficult to distinguish under strong shadow interference. Pavement repair traces may alter local texture continuity and lead to localization shifts, whereas background regions with damage-like textures may occasionally produce false-positive detections. These cases indicate that geometric adaptive enhancement cannot completely eliminate severe appearance ambiguity between damaged and undamaged pavement regions. Future work may therefore incorporate illumination-adaptive enhancement, hard-negative mining, boundary-aware representation, and temporal information from continuous road inspection videos to further improve robustness under challenging conditions.

## 5. Conclusions and Future Work

This paper proposes FasterGeo-YOLO, a task-oriented lightweight road damage detector that combines an efficiency-oriented FasterNet backbone, a modified distributional regression formulation with DFL, complementary P3/P4 deformable geometric adaptation, and a separate high-resolution refinement strategy. The revised analysis distinguishes the role of each component: DFL mainly strengthens the regression formulation, FasterNet improves practical inference efficiency while maintaining accuracy, P3/P4-DCN provides complementary adaptive sampling at different spatial resolutions, and the 800 × 800 stage further refines spatial detail without changing model parameters.

Under the fair 640 × 640 baseline comparison, FasterGeo-YOLO achieves P = 0.612, R = 0.540, mAP@0.5 = 0.567, and mAP@0.5:0.95 = 0.286. The subsequent 800 × 800 refinement produces the representative final result of P = 0.632, R = 0.538, mAP@0.5 = 0.580, and mAP@0.5:0.95 = 0.293. The controlled three-seed ablation at 640 × 640 further shows that the complete Step3 architecture reaches 0.5613 ± 0.0012 mAP@0.5 and 0.2831 ± 0.0004 mAP@0.5:0.95, compared with 0.5225 ± 0.0043 and 0.2598 ± 0.0014 for the exact DFL-free YOLO26n control. The resolution-only YOLO26n experiment also improves to 0.569/0.284 after 800 × 800 refinement, confirming that high resolution itself is beneficial; FasterGeo-YOLO remains higher at the matched 800 × 800 setting, so the final gain is not attributable solely to resolution.

Although the proposed method achieves improvements on the reported RDD2022 split, several limitations remain. First, D40 potholes remain difficult to localize precisely under high IoU thresholds because weak and irregular contours are not fully represented by rectangular boxes. Second, although RDD2022 includes images from multiple countries and visually diverse road environments, the present experiments use a fixed within-dataset split and therefore do not constitute a strict geographic generalization test. Country-wise or leave-one-country-out evaluation and independent external-dataset validation will be investigated in future work. Further work will also examine boundary-aware representations, real vehicle-mounted/edge-device deployment, and temporal information from continuous road inspection videos.

## Figures and Tables

**Table 1:** Dataset and Experimental Configuration.

Parameter	Setting
Dataset	RDD2022-cleaned; independent road damage images; fixed 7:3 train/validation split
Detection categories	D00, D10, D20, D40
Training/validation set	16,636/7131 images; 16,479 instances in validation; no separate untouched test subset
Stage 1	640 × 640, 100 epochs, batch size 32
Stage 2	800 × 800, 50 epochs, batch size 16
Optimizer	SGD with momentum is used for mainstream YOLO models; some legacy code repositories only support SGD; lr = 0.01, momentum = 0.9, weight decay = 0.0005.
Data augmentation	Mosaic = 1.0, HSV, random horizontal flipping, scale augmentation, and translation; Mosaic is disabled in the last 10 epochs; MixUp = 0.0, Copy-Paste = 0.0
External pretraining	YOLO-series models, road damage improved models, and FasterGeo-YOLO do not load external pretrained weights; Faster R-CNN, RetinaNet, and RT-DETR-L use COCO pretrained weights as representative detector references.
Runtime environment	Python 3.9.23, PyTorch 2.5.1+cu121, Ultralytics 8.4.38, NVIDIA RTX 4090 D
Random seed	Seeds 0, 1, and 2 for controlled multi-seed ablation; AMP = False; Deterministic = False

**Table 2:** Performance Comparison of Different Object Detection Methods on the RDD2022-cleaned Dataset.

Type	Model	External Pretraining	Input	P	R	mAP@0.5	mAP@0.5:0.95	Params/M	GFLOPs@640
Two-stage detector	Faster R-CNN R50-FPN	COCO	640	0.555	0.472	0.479	0.205	—	—
One-stage anchor-based detector	RetinaNet R50-FPN	COCO	640	0.580	0.438	0.431	0.187	—	—
Transformer-based detector	RT-DETR-L [36]	COCO	640	0.617	0.508	0.534	0.246	31.992	103.4
General lightweight detector	YOLOv5nu [31]	None	640	0.596	0.507	0.530	0.263	2.504	7.1
General lightweight detector	YOLOv8n [10]	None	640	0.600	0.517	0.543	0.274	3.006	8.1
General lightweight detector	YOLO11n [32]	None	640	0.621	0.507	0.546	0.275	2.583	6.3
Baseline of this paper	YOLO26n [33]	None	640	0.590	0.504	0.522	0.258	2.505	5.8
Improved road damage detection model	OBC-YOLOv8/YOLOv8-CA [34]	None	640	0.610	0.502	0.536	0.273	3.023	8.0
Improved road damage detection model	Improved YOLOv8s w/o DyHead [35]	None	640	0.569	0.486	0.506	0.254	2.440	6.3
Proposed method	FasterGeo-YOLO	None	640	0.612	0.540	0.567	0.286	2.762	7.059

Note: “None” indicates that no external pretrained weights are used. All models in the main baseline comparison are reported at 640 × 640 input resolution. Params and GFLOPs for Ultralytics-based models are reported using the unfused model definition when available; the proposed FasterGeo-YOLO contains 2.762 M parameters and requires 7.059 GFLOPs at 640 × 640. Faster R-CNN and RetinaNet complexity values are not reported because their profiling pipeline is not directly comparable. The COCO-pretrained Faster R-CNN, RetinaNet, and RT-DETR-L models are included as representative references for different detection paradigms rather than as strictly identical-pretraining comparisons.

**Table 3:** Controlled Sequential Ablation under Three Random Seeds (mean ± sample SD).

Step	Configuration	Input	Params/M	P	R	mAP@0.5	mAP@0.5:0.95	D40 mAP@0.5
Step0	Exact YOLO26n (DFL-free)	640	2.505	0.5943 ± 0.0069	0.4981 ± 0.0031	0.5225 ± 0.0043	0.2598 ± 0.0014	0.4034 ± 0.0084
StepH	YOLO26n + matched DFL	640	2.694	0.6176 ± 0.0034	0.5156 ± 0.0028	0.5496 ± 0.0026	0.2761 ± 0.0009	0.4353 ± 0.0064
Step1	+FasterNet redesign	640	2.642	0.6129 ± 0.0049	0.5207 ± 0.0063	0.5515 ± 0.0015	0.2767 ± 0.0008	0.4403 ± 0.0040
Step2	+P3-DCN	640	2.669	0.6143 ± 0.0039	0.5238 ± 0.0055	0.5539 ± 0.0029	0.2788 ± 0.0021	0.4473 ± 0.0092
Step3	+P3/P4-DCN	640	2.762	0.6218 ± 0.0047	0.5291 ± 0.0031	0.5613 ± 0.0012	0.2831 ± 0.0004	0.4548 ± 0.0069
Step4	+HiRes800 refinement	800	2.762	0.6315 ± 0.0049	0.5390 ± 0.0052	0.5750 ± 0.0026	0.2874 ± 0.0020	0.4692 ± 0.0046

**Table 4:** DCN Insertion-position Ablation at 640 × 640.

Model	Input	P	R	mAP@0.5	mAP@0.5:0.95	D40 mAP@0.5
FasterNet-only	640	0.615	0.520	0.554	0.281	0.441
FasterNet + P3-DCN	640	0.622	0.530	0.562	0.284	0.459
FasterNet + P4-DCN	640	0.618	0.525	0.557	0.283	0.444
FasterNet + P3/P4-DCN	640	0.612	0.540	0.567	0.286	0.466

Note: Table 4 is retained as a 640 × 640 insertion-position ablation to compare the relative effects of P3-only, P4-only, and joint P3/P4 deformable enhancement.

**Table 5:** Comparison of Structural Alternatives at 640 × 640.

Type	Method	mAP@0.5	mAP@0.5:0.95	D40 mAP@0.5	GFLOPs@640
Main structure	FasterNet + P3/P4-DCN	0.567	0.286	0.466	7.059
Loss function replacement	+NWD 0.1	0.559	0.282	0.450	7.059
Loss function replacement	+WIoU 0.3	0.563	0.284	0.456	7.059
Upsampling replacement	+DySample	0.565	0.288	0.454	7.0
High-resolution detection head	+P2 Head	0.564	0.288	0.450	10.4

Note: All methods in Table 5 are trained and validated at 640 × 640 input resolution. The table is used only to compare structural or loss-related alternatives. High-resolution refinement at 800 × 800 is discussed separately in the text and its actual 800 × 800 computational cost is reported in Section 4.6.

**Table 6:** Class-wise Detection Results of FasterGeo-YOLO.

Category	Meaning	P	R	mAP@0.5	mAP@0.5:0.95
D00	Longitudinal crack	0.643	0.551	0.596	0.329
D10	Transverse crack	0.618	0.561	0.582	0.285
D20	Alligator crack	**0.682**	**0.616**	**0.672**	**0.354**
D40	Pothole	0.586	0.425	0.472	0.206

Note: Bold values indicate the highest performance for each evaluation metric among the four road damage categories.

## Data Availability

The original data presented in the study are openly available in Figshare at https://doi.org/10.6084/m9.figshare.21431547.v1, reference number 21431547.

## References

[1] Maeda H., Sekimoto Y., Seto T., Kashiyama T., Omata H. (2018). Road damage detection and classification using deep neural networks with smartphone images. Comput.-Aided Civ. Infrastruct. Eng..

[2] Arya D.M., Maeda H., Ghosh S.K., Toshniwal D., Mraz A., Kashiyama T., Sekimoto Y. (2021). Deep learning-based road damage detection and classification for multiple countries. Autom. Constr..

[3] Arya D., Maeda H., Ghosh S.K., Toshniwal D., Omata H., Kashiyama T., Seto T., Mraz A., Sekimoto Y. (2021). RDD2020: An annotated image dataset for automatic road damage detection using deep learning. Data Brief..

[4] Arya D., Maeda H., Ghosh S.K., Toshniwal D., Sekimoto Y. (2024). RDD2022: A multi-national image dataset for automatic road damage detection. Geosci. Data J..

[5] Arya D., Maeda H., Ghosh S.K., Toshniwal D., Sekimoto Y. (2022). Crowdsensing-based road damage detection challenge 2022. arXiv.

[6] Redmon J., Divvala S., Girshick R., Farhadi A. (2016). You Only Look Once: Unified, real-time object detection. Proceedings of the IEEE Conference on Computer Vision and Pattern Recognition (CVPR).

[7] Redmon J., Farhadi A. (2018). YOLOv3: An incremental improvement. arXiv.

[8] Bochkovskiy A., Wang C.-Y., Liao H.-Y.M. (2020). YOLOv4: Optimal speed and accuracy of object detection. arXiv.

[9] Wang C.-Y., Bochkovskiy A., Liao H.-Y.M. (2023). YOLOv7: Trainable bag-of-freebies sets new state-of-the-art for real-time object detectors. Proceedings of the IEEE/CVF Conference on Computer Vision and Pattern Recognition (CVPR).

[10] Jocher G., Chaurasia A., Qiu J. (2023). Ultralytics YOLO, version 8.0.0.

[11] Sandler M., Howard A., Zhu M., Zhmoginov A., Chen L.-C. (2018). MobileNetV2: Inverted residuals and linear bottlenecks. Proceedings of the IEEE/CVF Conference on Computer Vision and Pattern Recognition (CVPR).

[12] Wang C.-Y., Liao H.-Y.M., Wu Y.-H., Chen P.-Y., Hsieh J.-W., Yeh I.-H. (2020). CSPNet: A new backbone that can enhance learning capability of CNN. Proceedings of the IEEE/CVF Conference on Computer Vision and Pattern Recognition Workshops (CVPRW).

[13] Tan M., Pang R., Le Q.V. (2020). EfficientDet: Scalable and efficient object detection. Proceedings of the IEEE/CVF Conference on Computer Vision and Pattern Recognition (CVPR).

[14] Chen J., Kao S.-H., He H., Zhuo W., Wen S., Lee C.-H., Chan S.-H. (2023). Run, Don’t Walk: Chasing higher FLOPS for faster neural networks. Proceedings of the IEEE/CVF Conference on Computer Vision and Pattern Recognition (CVPR).

[15] Lin T.-Y., Dollár P., Girshick R., He K., Hariharan B., Belongie S. (2017). Feature Pyramid Networks for object detection. Proceedings of the IEEE Conference on Computer Vision and Pattern Recognition (CVPR).

[16] Liu S., Qi L., Qin H., Shi J., Jia J. (2018). Path Aggregation Network for instance segmentation. Proceedings of the IEEE Conference on Computer Vision and Pattern Recognition (CVPR).

[17] Dai J., Qi H., Xiong Y., Li Y., Zhang G., Hu H., Wei Y. (2017). Deformable Convolutional Networks. Proceedings of the IEEE International Conference on Computer Vision (ICCV).

[18] Zhu X., Hu H., Lin S., Dai J. (2019). Deformable ConvNets v2: More deformable, better results. Proceedings of the IEEE/CVF Conference on Computer Vision and Pattern Recognition (CVPR).

[19] Hu J., Shen L., Sun G. (2018). Squeeze-and-Excitation Networks. Proceedings of the IEEE Conference on Computer Vision and Pattern Recognition (CVPR).

[20] Woo S., Park J., Lee J.-Y., Kweon I.S. (2018). CBAM: Convolutional Block Attention Module. Proceedings of the European Conference on Computer Vision (ECCV).

[21] Wang Q., Wu B., Zhu P., Li P., Zuo W., Hu Q. (2020). ECA-Net: Efficient Channel Attention for deep convolutional neural networks. Proceedings of the IEEE/CVF Conference on Computer Vision and Pattern Recognition (CVPR).

[22] Yang L., Zhang R.-Y., Li L., Xie X. (2021). SimAM: A simple, parameter-free attention module for convolutional neural networks. Proceedings of the 38th International Conference on Machine Learning (ICML).

[23] Wang J. (2021). A Normalized Gaussian Wasserstein Distance for tiny object detection. arXiv.

[24] Rezatofighi H., Tsoi N., Gwak J., Sadeghian A., Reid I., Savarese S. (2019). Generalized Intersection over Union: A metric and a loss for bounding box regression. Proceedings of the IEEE/CVF Conference on Computer Vision and Pattern Recognition (CVPR).

[25] Zheng Z., Wang P., Liu W., Li J., Ye R., Ren D. (2020). Distance-IoU loss: Faster and better learning for bounding box regression. Proceedings of the AAAI Conference on Artificial Intelligence (AAAI).

[26] Tong Z., Chen Y., Xu Z., Yu R. (2023). Wise-IoU: Bounding box regression loss with dynamic focusing mechanism. arXiv.

[27] Wang J., Chen K., Xu R., Liu Z., Loy C.C., Lin D. (2019). CARAFE: Content-Aware ReAssembly of FEatures. Proceedings of the IEEE/CVF International Conference on Computer Vision (ICCV).

[28] Liu W., Lu H., Fu H., Cao Z. (2023). Learning to upsample by learning to sample. Proceedings of the IEEE/CVF International Conference on Computer Vision (ICCV).

[29] Huang X., Chen H., Zhang W., Dai X., Li Y., Qing L., He X. (2026). AMIEOD: Adaptive Multi-Experts Image Enhancement for Object Detection in Low-Illumination Scenes. IEEE Trans. Multimed..

[30] Jia R., Wang X., Guo C. (2026). EFAF-YOLO: A Novel Enhanced Framework for Small-Object Detection. IEEE Geosci. Remote Sens. Lett..

[31] Jocher G., Chaurasia A., Stoken A., Borovec J., NanoCode012, Kwon Y., Michael K., TaoXie, Fang J., Imyhxy (2022). Ultralytics/Yolov5: V7.0—YOLOv5 SOTA Realtime Instance Segmentation. Zenodo.

[32] Khanam R., Hussain M. (2024). YOLOv11: An overview of the key architectural enhancements. arXiv.

[33] Sapkota R., Karkee M. (2025). Ultralytics YOLO evolution: An overview of YOLO26, YOLO11, YOLOv8 and YOLOv5 object detectors for computer vision and pattern recognition. arXiv.

[34] Zhang S., Liu Z., Wang K., Huang W., Li P. (2025). OBC-YOLOv8: An improved road damage detection model based on YOLOv8. PeerJ Comput. Sci..

[35] Wang J., Meng R., Huang Y., Zhou L., Huo L., Qiao Z., Niu C. (2024). Road defect detection based on improved YOLOv8s model. Sci. Rep..

[36] Zhao Y., Lv W., Xu S., Wei J., Wang G., Dang Q., Liu Y., Chen J. (2024). DETRs beat YOLOs on real-time object detection. Proceedings of the IEEE/CVF Conference on Computer Vision and Pattern Recognition (CVPR).

